# Mediastinal Lymph Node Metastases in Thyroid Cancer: Characteristics, Predictive Factors, and Prognosis

**DOI:** 10.1155/2017/1868165

**Published:** 2017-11-12

**Authors:** Ting-ting Zhang, Ning Qu, Jia-qian Hu, Rong-liang Shi, Duo Wen, Guo-hua Sun, Qing-hai Ji

**Affiliations:** ^1^Department of Head & Neck Surgery, Fudan University Shanghai Cancer Center, Shanghai 200032, China; ^2^Department of Oncology, Shanghai Medical College, Fudan University, Shanghai 200032, China

## Abstract

**Background:**

Mediastinal lymph node metastases (MLNM) have not been extensively studied. The aim of this study is to investigate the characteristics, predictive factors, and prognosis of MLNM in thyroid cancer.

**Methods:**

This is a retrospective study based on the thyroid cancer patients with MLNM at our institution from 2008 to 2015.

**Results:**

In total, 73 thyroid cancer patients with positive MLNM were included in this study. It contained sixty patients (82.2%) with papillary thyroid carcinoma (PTC), twelve (16.4%) with medullary thyroid carcinoma, and one (1.4%) with anaplastic thyroid carcinoma. Forty-eight patients had the surgery as initial treatment. Fifty-three (72.6%) patients remained disease-free, and fifteen (20.5%) developed a regional recurrence. Distant metastases occurred in four (5.5%) patients and five (6.8%) patients died. Five-year overall survival rate and disease-free survival (DFS) rate of the PTC patients for initial treatment are 95.4% and 77.2%, respectively. Extrathyroidal extension and multiple lymph nodes involved were associated with DFS in PTC patients.

**Conclusions:**

Initial therapeutic control is very important for the thyroid cancer patients. Extrathyroidal extension and multiple mediastinal lymph nodes involved were the influence factors of prognosis in the thyroid cancer patients with MLNM.

## 1. Introduction

Thyroid cancer commonly develops regional lymphatic metastases. Regional lymph nodes to which thyroid carcinoma can metastasize are classified into three regions: central, lateral, and mediastinal compartments [1]. Clinical characteristics and surgical management for both the central and the lateral lymph node metastases have been well described [2]. However, mediastinal lymph node metastases (MLNM) have not been extensively studied. Surgical treatment of MLNM requires a more extensive operation, which will potentially increase the risk of complications (e.g., pneumothorax and mediastinitis) and subsequently may affect their health-related quality of life. Therefore, careful selection of patients at high risk of MLNM to perform mediastinal lymph node dissection remains pivotal [3].

In this study, we analyzed the clinical data of the thyroid cancer patients who underwent mediastinal lymph node dissection at our institution between 2008 and 2015 and performed a systematic review of the literatures regarding MLNM in thyroid cancer to explore the characteristics, predictive factors, and prognosis for patients with mediastinal lymph node metastases.

## 2. Materials and Methods

### 2.1. Patient Selection

In total, 17,745 consecutive patients had surgery for thyroid cancer in the Department of Head & Neck Surgery at Fudan University Shanghai Cancer Center between January 2008 and December 2015. Among these, there were 17,125 cases with papillary thyroid carcinoma (PTC), 253 with follicular thyroid carcinoma (FTC), 239 with medullary thyroid carcinoma (MTC), 25 with anaplastic/undifferentiated thyroid carcinoma, and 103 with two pathologic types of thyroid carcinoma. Of 17,745 patients, 94 underwent the mediastinal lymph node dissection through transcervical or transsternal approach when mediastinal lymph node metastases were suspected according to the preoperative radiological examinations or intraoperative findings. One patient's postoperative pathological examination suggested that breast cancer and thyroid cancer simultaneously metastasized to the cervical and mediastinal lymph node, and thus this patient was excluded from the study. The patients included in our analysis had to have a minimum of 12 months of follow-up; thus, seven patients were excluded due to the deficiency of follow-up information. Moreover, 13 patients appeared negative to mediastinal nodes according to the postoperative pathology. Therefore, 73 thyroid carcinoma patients who met the criterion were enrolled finally in this cohort.

### 2.2. Initial Treatment and Follow-Up

All patients were diagnosed with thyroid carcinoma by fine-needle aspiration, frozen section, or the histopathologic report. Surgery was the main treatment for all patients. Mediastinal lymph node dissection was performed for the patients who had preoperative imaging demonstrating mediastinal lymph node metastases or who had clinically overt mediastinal nodes during the surgery. Mediastinal lymph node system is defined on both sides of the trachea, from the innominate artery and brachiocephalic vein down to the tracheal bifurcation within the anterior and posterior part of the mediastinum. Thyroid lobectomy was performed on 29 patients and total or near total thyroidectomy on 44 patients. Central lymph node dissection was performed on 9 patients, lateral lymph node dissection on 19 patients, central and lateral lymph node dissection on 43 patients, and mediastinal lymph node dissection alone on 2 patients who had received central and lateral lymph node dissection in other hospital with no lymph node metastasis in central and lateral regions suspected preoperatively. Among 73 patients, 7 patients (9.6%) were complicated by temporary hypocalcemia and one patient (1.4%) was complicated by permanent hypocalcemia. One patient (1.4%) showed temporary recurrent laryngeal nerve paralyses. No permanent recurrent laryngeal nerve paralyses, hemorrhage/hematomas, or chylous fistula were found. For the advanced differentiated thyroid cancer patients, according to the ATA guideline [2], adjuvant radiotherapy was recommended after total thyroidectomy.

CT/MRI of the mediastinum, ultrasound/CT of the neck, and the biomarker tests (the serum thyroglobulin for PTC and FTC after total thyroidectomy; the serum calcitonin for MTC) were routinely checked during follow-up. The cervical or mediastinal regional recurrence evidenced by imaging or pathological examinations, distant metastasis, or death was considered as an event of disease-free survival (DFS), while increasing thyroglobulin/calcitonin without solid tumor found by imaging examinations (including ultrasound, CT, MRI, or PET/CT) was not considered as an event of DFS. The event of relapse-free survival (RFS) was defined as the regional recurrence or death according to the same criterion as DFS, while distant metastasis was not as an event of RFS.

### 2.3. Clinical Data Collection

We reviewed all the patients' charts for data regarding basic information, clinical findings, preoperative diagnostic examinations, operative reports, histopathologic reports, and follow-up. This retrospective research was carried out in accordance with the ethical rules of Fudan University Shanghai Cancer Center.

To explore the influence factors of prognosis for the thyroid cancer patients with MLNM, several factors were analyzed in our study, including age, gender, pathological features of primary tumor (bilaterality, multifocality, and extrathyroid extension), the number of lymph nodes involved (central, lateral, and mediastinal compartment), lymph node invasion, T stage, distant metastasis, AJCC stage, and the approach of surgery. Pathological features of primary tumor, the number of lymph nodes involved, and lymph node invasion were diagnosed by experienced pathologists. Extrathyroidal extension was defined as gross tumor invasion of the strap muscles, larynx, trachea, esophagus, recurrent laryngeal nerve, mediastinal vessels, or carotid artery from the thyroid primary tumor site [4].

### 2.4. Literature Review

**Figure 1:**
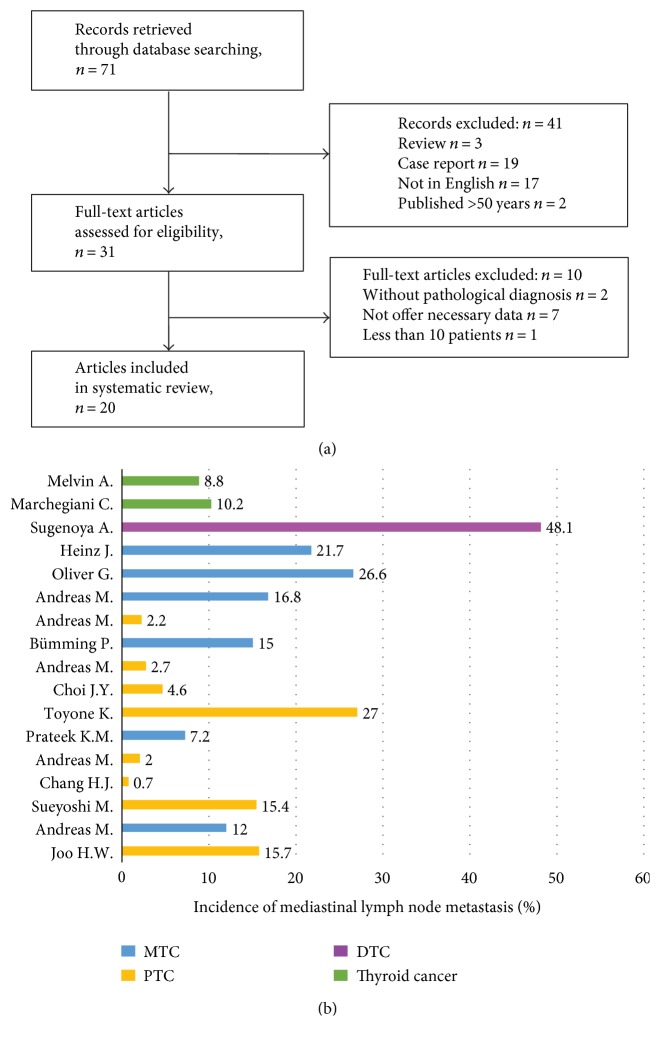
(a) The process of searching and screening the articles according to the inclusion/exclusion criterion; (b) the incidence of mediastinal lymph node metastasis in thyroid carcinoma previously reported.

A comprehensive literature search for studies published before December 2016 was performed in the PubMed and Web of Science databases referring the PRISMA-P (Preferred Reporting Items for Systematic Review and Meta-analysis Protocols) [5]. We used the following keywords as the search algorithm: mediastinal lymph node metastases/dissection and thyroid cancer. All of the reference lists from the main articles were inspected for additional eligible studies. In total, 71 studies were retrieved and 20 studies [6–25] were finally included in the analysis according to the inclusion/exclusion criterions (detailed process of selection was shown in Figure 1(a)).

### 2.5. Statistical Analysis

Statistical analyses were performed using SPSS ver. 22.0 (SPSS Inc., Chicago, IL, USA). Patient characteristics were compared using the chi-square test for categorical variables and Student's *t*-test for continuous variables. A *p* value less than 0.05 was considered significant. Overall survival, disease-specific survival, and disease-free survival were calculated using the Kaplan-Meier method. We performed a Cox regression multivariate analysis that included the predictive factors which had a significant relationship with prognosis in the univariate analysis.

## 3. Results

### 3.1. Characteristics of the Study Cohort

As shown in Table 1, a total of 73 thyroid cancer patients with MLNM were included in our research. There were 34 males and 39 females with a median age of 43 years (range 14 to 90 years).The average hospitalization time was 10.6 days. Histopathologically, there were 60 (82.2%) papillary thyroid carcinoma (PTC), 12 (16.4%) medullary thyroid carcinoma (MTC), and 1(1.4%) anaplastic thyroid carcinoma (ATC). Mediastinal lymph node dissection was carried out in 70 patients through a transcervical approach and 3 through a midline sternotomy. Among the 73 patients, 48 patients had the surgery as initial treatment, while the other 25 patients had the reoperation due to the recurrence of thyroid cancer. According to the pathological examinations, 50 patients (68.5%) were also accompanied with positive central and lateral lymph nodes, 21 patients (28.8%) only with positive lateral lymph nodes, and 2 patients (2.7%) only with positive mediastinal lymph nodes. In the 12 MTC patients, 11 patients presented preoperative increased serum calcitonin levels (>3000 pg/ml), and serum calcitonin levels were obviously decreased after surgery. Another MTC patient's serum calcitonin level was 47.46 pg/ml preoperative and decreased to 21.23 pg/ml after surgery. In the 60 PTC patients, 45 patients received total thyroidectomy. All of 45 patients' serum thyroglobulin levels were obviously decreased after surgery, and most of them decreased to 0–10 ng/ml levels.

### 3.2. Follow-Up and Oncologic Outcomes

Follow-up was ended on December 1st, 2016. The mean follow-up was 42.8 months (range 12 to 101 months). 53 (72.6%) of 73 patients with positive MLNM were alive and remain disease-free at the end of follow-up. Fifteen (20.5%) developed regional recurrence. Lung metastasis before surgery occurred in four patients. Four patients (5.5%) had postoperative distant metastasis—two with lung metastasis, one with bone metastasis, and one with lung and brain metastasis. During the follow-up, three patients (4.1%) died of thyroid cancer and two (1.4%) of other reasons.

### 3.3. Characteristics and Outcomes of the PTC Cohort for Initial Treatment

Difference was confirmed in prognosis between PTC and MTC. Besides, the cohort who had the surgery as initial treatment could reveal the characteristics and prognosis of the thyroid cancer patients with MLNM better. Thus, we analyzed the PTC cohort at initial treatment separately.

In total, 47 PTC had surgery as the initial treatment and proved with positive mediastinal lymph nodes in our research. The characteristics and prognosis of the cohort were shown in Table 2. The patients all had the surgery through the transcervical approach and appeared N1b. Five-year overall survival rate, disease-free survival rate, and relapse-free survival rate is 95.4%, 77.2%, and 79%, respectively.

### 3.4. The Influence Factors of the Prognosis of the PTC Patients for Initial Treatment

No significant factor was found to be associated with overall survival (OS) of the PTC patients with MLNM. In the Cox univariate and multivariate regression analysis, extrathyroidal extension (HR = 8.06, 95% CI 1.45–44.87, *p* < 0.05) and the number of mediastinal lymph nodes involved (HR = 1.83, 95% CI 1.01–3.30, *p* < 0.05) were associated with the DFS and RFS of the PTC patients with MLNM. Age, gender, multifocality, bilaterality, tumor size, and lymph node invasion were not associated with DFS or RFS in the analysis (*p* > 0.05) (Table 3).

### 3.5. Literature Review

There have been only limited research reports about MLNM in thyroid cancer. Thus, we applied the method of systematic literature review to investigate the incidence, predictive factors, and prognosis of mediastinal lymph node metastases in thyroid cancer. Through comprehensive search and reasonable scientific screening, 20 studies [6–25] were included finally (Table 4).

The incidence of mediastinal lymph node metastases in thyroid cancer was reported to range from 0.7% to 48.1% (Figure 1(b)) [6–9, 11–15, 17, 20–25]. As reported, the incidence of mediastinal lymph node metastases was 7.2–26.6% in MTC [8, 12, 17, 20, 21, 23] and 0.7–27% in PTC [6, 7, 9, 11, 13–15, 20]. The patients underwent mediastinal lymph node dissection only when MLNM was highly suspected in all of the studies except two studies. Woo et al. [6] and Kikumori and Imai [13] reported that the incidence of MLNM in PTC was 15.7% and 27% in the patients who had prophylactic mediastinal lymph node dissection, which was higher than the incidence of 0.7%–15.4% in other studies.

Many predictive factors for MLNM were discussed in previous studies [6, 7, 10, 11, 14–16, 18, 20, 22, 23], but most of them are still controversial (Table 5). Nevertheless, it mainly came to an accordant conclusion that the patients with bilateral cervical metastasis [10, 15, 18, 22, 23] or with distant metastasis [10, 11, 15, 18] and the patients who underwent reoperation [6, 18, 20] had increased risk of MLNM. And the predictive factors discussed in each study were shown in the supporting information.

There are totally five studies [7, 10, 13, 16, 19] revealing the prognosis of the patients with MLNM. Liu et al. [10] demonstrated that the incidences of death, recurrence, and distant metastasis were 12.6%, 14.3%, and 16.8%, respectively, based on 119 PTC cases. Kikumori and Imai [13] reported that, in a series of 184 PTC patients, the incidences of death, recurrence, and distant metastasis were 9.2%, 4.9%, and 0.5%, respectively, and the disease-specific survival (DSS) was 97.9%. In the series of 31 patients with thyroid cancer metastasizing to mediastinum, no death or distant metastasis occurred but 9.7% of patients developed recurrence during follow-up [16]. Khoo and Freeman [19] followed up 30 PTC patients with MLNM and investigated that there were no cases of death or recurrence, but 3.3% of patients developed distant metastasis. Moritani [7] specially investigated the impact of mediastinal metastases on the prognosis of PTC based on the mean 10.5-year follow-up of 488 patients. They found significant differences between patients with mediastinal metastases dissected by either transcervical/sternotomy and patients without metastases in DFS. But the differences in OS between patients with mediastinal metastases dissected via the transcervical approach and patients without metastases were not significant (*p* > 0.05). The OS of patients with mediastinal metastases dissected by sternotomy was significantly poorer (66.7%). The study also demonstrated that the age 45 years or older, male gender, extrathyroidal extension, poor differentiation, contralateral node metastasis, and mediastinal metastasis were independent predictive factors for RFS in PTC patients.

## 4. Discussion

Thyroid cancer has a strong propensity to metastasize to regional lymph nodes, and the incidence of both occult and overt nodal metastases is high [1]. The most common region where thyroid cancer metastasizes is central lymph nodes, followed by lateral and mediastinal lymph nodes [1, 20]. Central lymph node dissection was a widely accepted procedure and currently performed routinely in the treatment of thyroid cancer in many institutions [2]. There are lots of researches focused on the lateral lymph node metastases so far. However, the indications and extent of the mediastinal lymph node dissection in thyroid carcinoma are not clearly defined. Nevertheless, mediastinal lymph node dissection would be accompanied with greater surgical invasiveness and stress, especially through a transsternal procedure [26, 27]. There were two approaches of mediastinal lymphadenectomy: transcervical and transsternal procedures. Because complete sternal split may result in an increased morbidity, careful selection of patients at high risk of MLNM remains important. Therefore, it is meaningful to investigate the characteristics, predictive factors, and prognosis of mediastinal lymph node metastases in thyroid cancer.

As a matter of principle at our institution, the selection of patients for mediastinal lymph node dissection, especially for a transsternal procedure, requires demonstration of mediastinal lymph node metastases on preoperative imaging examinations, including CT, MRI, PET/CT, and so on. However, it is difficult to evaluate the mediastinal lymph node metastases accurately only depending on clinical examinations. Among the 94 patients with highly suspected MLNM who received mediastinal lymph node dissection at our institution, 13 (13.8%) patients appeared pathologically negative to mediastinal lymph nodes. Besides, lateral and central node metastases are frequently detected in papillary carcinoma, even if they are not detectable by preoperative imaging tests. Similarly, there would be some patients with occult mediastinal nodal metastases but without radiologic evidence. Thus, it is necessary to explore the indications of the mediastinal lymph node dissection with thyroid carcinoma.

Mediastinal lymph node metastases are generally thought to spread from paratracheal or pretracheal lymph nodes and lateral lymph nodes through the lymph circulation [22, 25]. However, metastasis to the mediastinal region directly from the primary tumor without lateral or central node metastasis was also demonstrated [20]. Among the 73 patients in our study, 50 patients (68.5%) were also accompanied with positive central and lateral lymph nodes, 21 patients (28.8%) only with positive lateral lymph nodes, and 2 patients (2.7%) only with positive mediastinal lymph nodes. However, the two cases only with positive MLNM had received central and lateral node dissection in other hospital and no recurrence was suspected in central and lateral regions preoperatively. Previous studies [11, 20, 22, 23] showed that contralateral lateral node metastasis was significantly correlated with mediastinal node metastases.

Bilateral cervical metastasis, distant metastasis, and reoperation were proved as the predictive factors of mediastinal metastasis in different pathologic types of thyroid cancer in several studies. However, the predictive value of age, pathologic subtypes, tumor size, T stage, extrathyroidal extension, lymphovascular invasion, central neck involvement, and lateral neck involvement were still controversial. Machens and Dralle [8] described the time-dependent change of the incidence of mediastinal lymph node metastases in thyroid cancer. The incidence of mediastinal lymph node metastases was down to 6% in the cohort of MTC patients during 2011–2015 compared to 21% during 1995–2000. They thought that the shift of incidence was attributed to the early diagnosis and increasing therapeutic control. Besides, reoperation was proved as a predictive factor of mediastinal lymph node metastases [6, 18, 20]. Initial therapeutic control is very important for the patient with thyroid cancer. And the patients with bilateral cervical metastasis or distant metastasis probably require further examination to determine whether mediastinal lymph node metastases exist.

In this study, extrathyroidal extension (HR = 8.06, 95% CI 1.45–44.87, *p* < 0.05) and the number of mediastinal lymph nodes involved (HR = 1.83, 95% CI 1.01–3.30, *p* < 0.05) were associated with DFS and RFS, but neither of them impacted OS or DSS. Moritani [7] demonstrated that the age 45 years or older, male gender, extrathyroidal extension, poor differentiation, contralateral node metastasis, and mediastinal metastasis were independent predictive factors for RFS in PTC patients. The number of lymph nodes involved and the largest tumor dimension within these nodes are important prognostic factors according to the ATA guidelines [2]. Further studies are necessary to explore the influence factors of prognosis of the thyroid cancer patients with MLNM.

The main limitations of this study are the relatively short follow-up time and the small sample size. Longer follow-up would show the prognostic characteristics of thyroid cancer patients with MLNM better due to the favorable prognosis of thyroid cancer. Small sample size resulted in inadequate events to perform multivariable analysis; thus, the predictive factors of MLNM and influence factors of prognosis could not be analyzed further. Moreover, a larger cohort is necessary to explore the predictive factors for mediastinal lymph node metastases and influence factors of prognosis better.

## 5. Conclusions

Mediastinal lymph node dissection is an effective treatment of MLNM in thyroid carcinoma no matter for initial treatment or reoperation of recurrent lesions. Careful selection of patients at high risk of MLNM to perform mediastinal lymph node dissection remains important. Initial therapeutic control is very important for the patients with thyroid cancer. The prognosis of the thyroid cancer patients with MLNM conforms to the prognostic trend of thyroid cancer reported previously. Bilateral cervical metastasis, distant metastasis, and reoperation were the predictive factors of mediastinal metastasis in thyroid cancer. Extrathyroidal extension and multiple mediastinal lymph nodes involved were the main influence factors of prognosis in the patients with thyroid cancer metastasizing to mediastinum.

## Figures and Tables

**Table 1:** The baseline characteristics of the patients included.

	Total patients (*n* = 73)	Initial surgery (*n* = 48)	Reoperation (*n* = 25)	*p* value
Gender (*n*)				
Male	34	24	10	0.520
Female	39	24	15	
Age (y) median (range)	43 (14–90)	42.5 (14–66)	44 (21–90)	0.709
Hospitalization time (d) mean ± SD	10.6 ± 5.1	10.5 ± 5.4	10.8 ± 4.8	0.849
Approaches of MLND				
Transcervical	70	46	24	0.973
Transsternal	3	2	1	
Follow-up time (m) mean (range)	42.8 (12–101)	39.4 (12–101)	46.9 (12–86)	0.246
Histopathology (*n*)				
PTC	60	47	13	0.658
MTC	12	0	12	
ATC	1	1	0	
Prognosis *n* (%)				1.000
Disease-free	53 (72.6)	35 (72.9)	18 (72.0)	
Recurrence	15 (20.5)	11 (22.9)	4 (16.0)	
Distant metastasis	4 (5.5)	3 (6.3)	1 (4.0)	
Death	5 (6.8)	3 (6.3)	2 (8.0)	
Disease-specific death	3 (4.1)	2 (4.2)	1 (4.0)	

PTC: papillary thyroid carcinoma; MTC: medullary thyroid carcinoma; ATC: anaplastic thyroid carcinoma; SD: standard deviation; MLNM: mediastinal lymph node metastases.

**Table 2:** Characteristics of the PTC patients for initial treatment.

Age (y) mean (range)	PTC for initial treatment (*n* = 47)	
	40 (14–66)	
	Number	Percent (%)
Gender		
Male	23	48.9
Female	24	51.1
Approaches of MLND		
Transcervical	47	100
Transsternal	0	0
Bilaterality	18	38.3
Multifocality	20	42.6
ETE	14	29.8
Median number of lymph nodes involved (*n*)		
Central	4	—
Lateral	8	—
Mediastinal	1	—
Lymph node invasion	3	6.4
T stage		
T1a	5	10.6
T1b	15	31.9
T2	11	23.4
T3	7	14.9
T4	9	19.1
M stage		
M0	44	93.6
M1	3	6.4
AJCC stage		
I	26	55.3
IV	21	44.7
Surgery		
TT or nTT	29	61.7
Unilateral lobectomy	18	38.3
Lateral and central LND	35	74.5
Central LND	12	25.5
Prognosis		
Disease-free	38	80.9
Recurrence	7	14.9
Distant metastasis	1	2.1
Death	2	4.3
Disease-specific death	1	2.1

MLNM: mediastinal lymph node dissection; ETE: extrathyroidal extension; TT: total thyroidectomy; nTT: near total thyroidectomy; LND: lymph node dissection.

**Table 3:** Cox univariate and multivariate analysis for the effect factors of prognosis of the PTC patients for initial treatment.

Factors	DFS					RFS				
	Univariate	Multivariate				Univariate	Multivariate			
	*p*	*p*	HR	95%CI		*p*	*p*	HR	95%CI	
				Lower	Up				Lower	Up
Age	0.953	0.472	0.98	0.92	1.04	0.638	0.472	0.98	0.92	1.04
Multifocality	0.715	0.627	1.53	0.27	8.53	0.958	0.627	1.53	0.27	8.53
ETE	0.069	0.017^∗^	8.06	1.45	44.87	0.038^∗^	0.017^∗^	8.06	1.45	44.87
Number of mediastinal lymph nodes involved	0.057	0.045^∗^	1.83	1.01	3.30	0.057	0.045^∗^	1.83	1.01	3.30

ETE: extrathyroidal extension. ^∗^*p* < 0.05.

**Table 4:** The information about the studies included in the systemic review.

Author	Country	Year	Sample	Type	Treatment	Approach of MLND	CLND (%)	MLND (%)	LLND (%)	Surgery (IN:RE)	Age (y)	Gender (F:M)	Incidence (%)	Predictive factors
Moritani [7]	Japan	2016	488	PTC	—	Transcervical (76%) + sternotomy (24%)	100	15.40	50.80	488 : 0	51	374 : 114	15.40	Age (>45), number of central node metastases
Woo et al. [6]	Korea	2016	217	PTC	TT	Transcervical	100	100	17.10	194 : 23	48.7	181 : 36	15.7	Revision surgery, pretracheal pN(+), and multiple lateral metastases
Machens and Dralle [8]	Germany	2016	600	MTC	TT	Sternotomy	92	14	83	322 : 278	52.8	233 : 47	12	—
Chang et al. [9]	Korea	2015	5556	PTC	TT	Not shown	100	0.92	15.60	5556 : 0	46.2	4385 : 1171	0.7	Right para-oesophageal lymph node metastasis
Liu et al. [10]	China	2013	119	PTC	TT lobectomy	Transcervical (71.4%) + sternotomy (26.1%)	100	100	86.60	75 : 44	—	69 : 50	—	Bilateral metastasis and distant metastasis
Machens and Dralle [11]	Germany	2012	972	PTC	TT	Sternotomy	100	4	69	282 : 690	43	661 : 311	2	Distant metastasis
Mehrota et al. [12]	India	2011	71	MTC	TT + nTT	Sternotomy	68	7.20	62.30	62 : 9	39.9	44 : 27	7.2	—
Kikumori and Imai [13]	Japan	2011	184	PTC	TT	Sternotomy (17.9%) + transcervical (82.1%)	100	100	100	184 : 0	44.2	—	27	—
Choi et al. [14]	Korea	2011	195	PTC	TT + nTT + lobectomy	Transcervical	100	100	7.70	195 : 0	47.7	165 : 30	4.6	Tumor size (1.5 cm), lateral metastasis, and lymphovascular invasion
Machens and Dralle [15]	Germany	2009	392	PTC	TT + nTT + lobectomy	Sternotomy	91.3	7.40	90.30	202 : 190	39.6	241 : 151	2.7	Age, poor differentiation, ETE, bilateral metastasis, number of lymph nodes, and distant metastasis
Ducic and Oxford [16]	USA	2009	31	Thyroid carcinoma	TT	Transcervical	100	100	—	31 : 0	53.4	21 : 10	—	ETE, cervical metastasis
Bümming et al. [17]	Sweden	2008	20	MTC	TT	Sternotomy	100	15	100	20 : 0	47.5	15 : 5	15	—
Machens et al. [18]	Germany	2004	83	MTC	TT	Sternotomy	100	100%	99	25 : 58	44.7	48 : 35	—	T stage, ETE, bilateral metastasis, distant metastasis, calcitonin level, reoperation
Khoo and Freeman [19]	Canada	2002	30	PTC	TT	Transcervical	100	100%	93.30	30 : 0	42	24 : 6	—	—
Machens et al. [20]	Germany	2002	296	PTC (45.3%) MTC (54.7%)	TT	Sternotomy	100	PTC 8.2 MTC 25.3	PTC 35.8 MTC 77.2	100 : 196	—	—	PTC 2.2 MTC 16.8	Pathology, reoperation, and >35 mm
Gimm et al. [21]	Germany	1998	64	MTC	TT	Transcervical	100	79.70	90.60	27 : 37	49.6	40 : 24	26.60	—
Sugenoya et al. [22]	Japan	1993	21	DTC	TT	Partial sternotomy	100	100	100	21 : 0	52.4	10 : 11	48.1	Tumor size and cervical metastases
Buhr et al. [23]	Germany	1993	23	MTC	TT	Sternotomy (8.7%) + transcervical (91.3%)	100	100	100	0 : 23	43	11 : 12	21.70	T stage and bilateral metastasis
Marchegiani et al. [24]	Italian	1985	322	Thyroid carcinoma	TT	Transcervical	100	10.20	40.70	322 : 0	—	—	10.20	—
Block et al. [25]	America	1972	284	Thyroid carcinoma	—	Transcervical	100	8.80	100	17 : 8	—	—	8.80	—

CLND: central lymph node dissection; LLND: lateral lymph node dissection; MLND: mediastinal lymph node dissection; MLNM: mediastinal lymph node dissection; ETE: extrathyroidal extension; TT: total thyroidectomy; nTT: near total thyroidectomy.

**Table 5:** Predictive factors reported in previous studies.

Author	Type	Age	Cytological type	Tumor size	T stage	ETE	Lymphovascular invasion	Reoperation	Central neck involvement	Lateral neck involvement	Bilateral cervical metastasis	Distant metastasis
Woo et al. [6]	PTC		X	X	X	X	X	√	√	√		
Moritani [7]	PTC	√							√			
Liu et al. [10]	PTC	X			X				X	X	√	√
Machens and Dralle [11]	PTC											√
Choi et al. [14]	PTC			√			√			√		
Machens and Dralle [15]	PTC	√	√	X		√					√	√
Machens et al. [18]	MTC					√		√			√	√
Buhr et al. [23]	MTC				√						√	
Sugenoya et al. [22]	DTC				√						√	
Ducic and Oxford [16]	Thyroid					√			√			
Machens et al. [20]	PTC, MTC		√	√				√				

√ stands for the factor that was proved to have predictive value of the mediastinal lymph node metastasis in thyroid carcinoma; X stands for the factor that was proved to have no predictive value of the mediastinal lymph node metastasis in thyroid carcinoma. ETE: extrathyroidal extension.
